# Barker on Puerpeial Diseases

**Published:** 1874-04

**Authors:** 


THE PUERPERAL DISEASES. *Clinical Lectures delivered at Bellevue Hospital.* By Fordyce Barker, M.D., Clinical Professor of Midwifery and the Diseases of Women, in the Bellevue Hospital Medical College, etc. New York: D. Appleton & Co., 1874. Octavo, cloth, pp. 526. Price $5.00.

Those of our readers who have kept pace with the medical journals since the war, will be more or less familiar with the views of Prof. Barker upon many of the topics treated in the work before us. For the benefit of those who have not, we will endeavor to give an idea of its contents, and of the views of its author upon certain of its salient points.

After-pains are attributed to coagula within the uterus, and tully's powder, a formula for which is given, is recommended. Secondary hæmorrhage, *i.e.*, occurring from six hours after delivery up to the end of the month, receives a full and proper consideration, which is not given it in our text-books upon midwifery. The use of castor oil as a purgative for the lying-in chamber, especially if there be tendency to piles, is condemned, and the treatment of hemorrhoids by aloes recommended. The views expressed upon these points are peculiar to Prof. B. Support to the perineum is commended as a safeguard against laceration, and the use of the forceps, under certain circumstances, suggested with the same view; and also anæsthetics.

In albuminuria *antepartum* venesection, hydragogue cathartics, iron and premature labor are suggested. "I now rarely encounter puerperal convulsions, when the previous detection of albuminuria has led me to be particularly apprehensive of their occurrence. Indeed, I will go further, and say that, in most cases, where any of the predisposing causes that I have mentioned are discovered sufficiently early, they may be successfully treated, and convulsions will occur only in a small percentage." The prophylactic measures advised are venesection, brisk purgation, chloroform, hypodermic morphia and speedy delivery. Milk fever, mastitis and abcess are well treated. In puerperal mania, "I have never seen opium in any doses cut short the attack, although I have often known it to be tried." Chloroform has always failed in his hands, but he "finds the chloral-hydrate of immense value." He gives fifteen or twenty grains, well diluted, every two hours until sleep is induced.

"While we know that phlegmasia dolens occurs in the puerperal state and in association with diseases which cause inopexia, and that its most uniform autopsical lesion is venous thrombosis, we are still as ignorant of its real pathological nature as we are of rheumatism and many other diseases." In puerperal phlebitis veratrum, opium and vaginal carbolized injections are advised. The formula for injection using "a tumblerful of warm water," seems to us entirely inadequate for properly cleansing and disinfecting the parts. Puerperal metritis and puerperal peritonitis are each separately considered. In the latter affection reliance is placed in opium, veratrum viride, turpentine stupes, quinine, stimulants and carbolized injections; the latter again in half-pint instead of half-gallon quantities. Pelvic peritonitis, pelvic cellulitis and pelvic abcess receive due consideration. Quinine and alcohol are the remedies of chief reliance. In puerperal septicæmia and pyæmia antiseptic injections and scrupulous cleanliness are enjoined, and "the chief indication is to sustain the vital powers; or, in other words, to keep the patient alive while the system is making an effort to get rid of the poison and to recover from its effects;" quinine, veratrum, alcohol, etc., are advised. The author does not regard puerperal pyæmia as so fatal a disease as is generally represented. Quinine and alcohol are his remedies.

Puerperal fever is separately considered. He thinks it is probably due to some local cause, and "the evidence is overwhelming and conclusive that it is a contagious disease." "It is a distinct disease from the febrile reaction of inflammation of any of the tissues of the puerperal woman; * * and the anatomical lesions bear the same relation to this disease as the pustules on the cutaneous surface bear to small-pox," etc. Remedies: veratrum, opium, quinine, alcohol and antiseptic washes.

We think that Prof. Barker has made a valuable addition to our medical literature in the handsome volume we have so imperfectly sketched. His views we think very sound, very conservative and very safe.

